# Supplementary figures and images for: Study protocol: A systematic review and meta-analysis regarding the influence of coagulopathy and immune activation on new onset atrial fibrillation in patients with sepsis
Source: PLoS One. 2023 Sep 8;18(9):e0290963. doi: 10.1371/journal.pone.0290963 (PMC10490925; doi:10.1371/journal.pone.0290963)

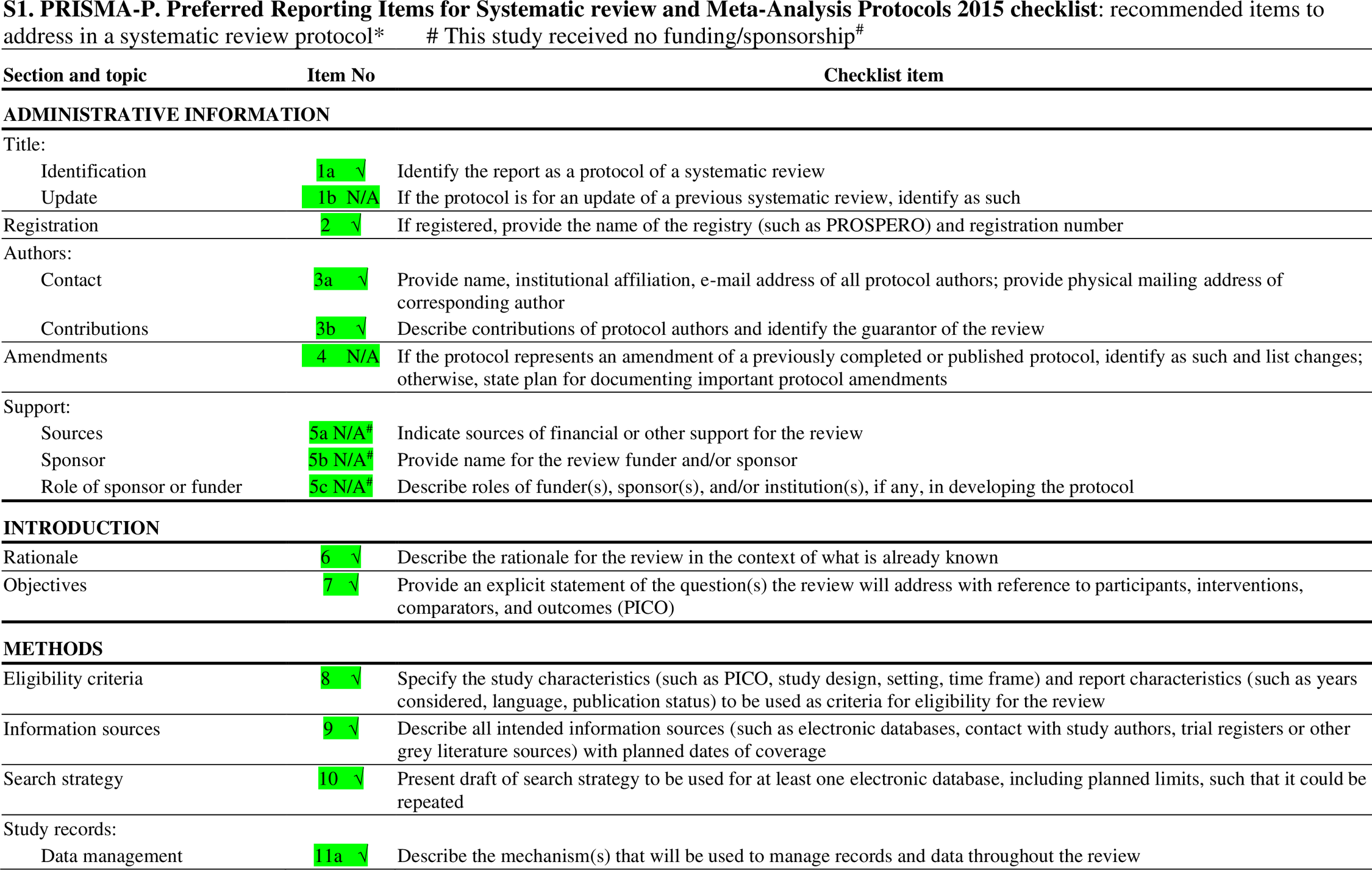

Supplement: S1 Fig — Preferred Reporting Items for Systematic review and Meta-Analysis Protocols 2015 checklist completed for this systematic review study protocol. (TIF) [file pone.0290963.s001.tif]
