# Supplementary material for: Study protocol: A systematic review and meta-analysis regarding the influence of coagulopathy and immune activation on new onset atrial fibrillation in patients with sepsis
Source: PLoS One. 2023 Sep 8;18(9):e0290963. doi: 10.1371/journal.pone.0290963 (PMC10490925; doi:10.1371/journal.pone.0290963)
Supplement: Attachment — Submitted filename: Response to Reviewers.docx [file pone.0290963.s002.docx]

1) Has a research librarian been involved in the development of the search strategy? If so, this should be mentioned in the methods. If not then this is a significant limitation. They are most likely to be up to date with recent changes in MESH terminology and database scope.

A research librarian was involved in the development of our search strategy and retrieving all relevant articles. We have updated our manuscript to reflect this and have also include the librarian in our acknowledgements.

2) I would expect to see a much clearer search strategy prior to consideration of publication, such as that outlined here: https://links.lww.com/CCM/E201. It needs to be more clear which terms are MESH, and precisely how the concepts will be combined. It should be specified which terms are being searched for in title only, or title and abstract, or title, abstract and author keywords, etc. I don’t believe it is sufficient to state “Boolean operators (OR, AND)... are to be utilised throughout the search strategy“ – these should be clarified.

We thank the reviewers for their suggestion and have included our complete search strategy within Table 1. We have included our search strategy for each database searched.

3) The aim / inclusion criteria feel unfocussed. What exactly is meant by “Studies that describe AF/NOAF in relation to sepsis/ critical illness/intensive care”? Does this include any study of NOAF in the ICU? Or only studies of patients with sepsis in the ICU? Or only studies that report sepsis-related biomarkers in both NOAF and non-NOAF groups? In your aim you state “We aim to analyse the link between coagulopathy and immune activation, and the development of NOAF in septic patients”. Is this therefore a systematic review of sepsis / inflammatory / coagulation-related risk factors (biomarkers only, or vital signs as well?) for NOAF in patients admitted to an ICU? The search strategy should be tailored to these specific criteria.

We have significantly reworded these sections of the manuscript to ensure clarity. Our aim is to assess if individual coagulation or inflammatory biomarkers can be utilised to identify patients with sepsis that are at risk of developing NOAF. We wish to retrieve articles that report biomarkers in patients admitted with sepsis who develop and do not develop NOAF. We are limiting our review to biomarkers only and therefore not including physio markers such as vital signs. Physio markers as risk factors for NOAF has been extensively reported elsewhere and will therefore not be the focus of this review. We will use the most recent sepsis 3 definition to define our sepsis population. We anticipate that some studies will predate the sepsis 3 consensus definition (2016) however, the definition is broader than previous definitions so we are confident that all relevant papers will be included in this definition.

4) The authors may which to re-word their approach to the I2 statistic “with I2 used to interpret the percentage of variance across the studies.” – more accurately it would estimate the percentage of total variability due to between-study heterogeneity. I would advise caution when using the I2 statistic to categorise the amount of heterogeneity (see https://doi.org/10.1002/jrsm.1230 for more details).

The I2 statistic has been reworded to more accurately represent its use, see updated manuscript.

Minor
Introduction: The second line is a little jarring. It introduces both the concept of “new-onset”, and the higher incidence in the subgroup of patients with sepsis at the same time, and starts the sentence with “in particular” which doesn’t really work.

We thank the reviewers for their comments. We have re-written the introduction to improve clarity of the manuscript. We have also made it clear that new onset atrial fibrillation is a subgroup of atrial fibrillation that is common in sepsis.

The manuscript would benefit from a thorough review from one of the senior authors to tidy up some of the language. Examples below:
• Introduction line 7: I’d replace “inciting” with “conferring”

Inciting has been replaced with conferring as requested and the manuscript has been re-reviewed by a senior author for clarity.

• Aim: You have “hypothesized” with a z, but “analyse” with an s. Please standardise / standardize. Suggest a z for this international journal.

Language has been converted to standard American English, see manuscript.

• Protocol design: You state “in accordance with the Preferred Reporting Items for Systematic Reviews and Meta-Analyses guideline (PRISMA-P)” – you have missed the word “Protocols” – same in “strengths and limitations”.

Protocols has been added to all mentions of PRISMA-P.

• Data sourcing: phrases like “Utilising our research question…” are unnecessary in this context.

Unnecessary phrases such as “Utilising our research question…” have been removed.

• Clear language: Suggest replacing most instances of “shall” with “will”, e.g. in “We shall assess risk of bias…”

Instances of shall have been replaced with will for clarity.

• Clear language: In the abstract, I think “management and treatment strategies for NOAF” could be simplified to “management strategies for NOAF”.

“management and treatment strategies for NOAF” has been simplified to “management strategies for NOAF”.

• “Septic patients” is a little labelling. I would encourage a change to “patients with sepsis” throughout the manuscript.

Patients with sepsis is now used throughout the document.
